# CXCR2 Is Deregulated in ALS Spinal Cord and Its Activation Triggers Apoptosis in Motor Neuron-Like Cells Overexpressing hSOD1-G93A

**DOI:** 10.3390/cells12141813

**Published:** 2023-07-09

**Authors:** Valentina La Cognata, Agata Grazia D’Amico, Grazia Maugeri, Giovanna Morello, Maria Guarnaccia, Benedetta Magrì, Eleonora Aronica, Velia D’Agata, Sebastiano Cavallaro

**Affiliations:** 1 Institute for Biomedical Research and Innovation, National Research Council, 95126 Catania, Italy; 2 Section of Human Anatomy and Histology, Department of Biomedical and Biotechnological Sciences, University of Catania, 95123 Catania, Italy; 3 Department of (Neuro) Pathology, Amsterdam UMC, University of Amsterdam, Amsterdam Neuroscience, Meibergdreef 9, 1105 Amsterdam, The Netherlands

**Keywords:** CXCR2, IL-8, CXCL8, CXCL1, CXCL2, amyotrophic lateral sclerosis, neurodegeneration, reparixin, GROα, MIP2α, inflammation

## Abstract

Amyotrophic lateral sclerosis (ALS) is a multifactorial neurodegenerative disease characterized by progressive depletion of motor neurons (MNs). Recent evidence suggests a role in ALS pathology for the C-X-C motif chemokine receptor 2 (CXCR2), whose expression was found increased at both mRNA and protein level in cortical neurons of sporadic ALS patients. Previous findings also showed that the receptor inhibition is able to prevent iPSC-derived MNs degeneration in vitro and improve neuromuscular function in SOD1-G93A mice. Here, by performing transcriptional analysis and immunofluorescence studies, we detailed the increased expression and localization of CXCR2 and its main ligand CXCL8 in the human lumbar spinal cord of sporadic ALS patients. We further investigated the functional role of CXCR2/ligands axis in NSC-34 motor neuron-like cells expressing human wild-type (WT) or mutant (G93A) SOD1. A significant expression of CXCR2 was found in doxycycline-induced G93A-SOD1-expressing cells, but not in WT cells. In vitro assays showed CXCR2 activation by GROα and MIP2α, two murine endogenous ligands and functional homologs of CXCL8, reduces cellular viability and triggers apoptosis in a dose dependent manner, while treatment with reparixin, a non-competitive allosteric CXCR2 inhibitor, effectively counteracts GROα and MIP2α toxicity, significantly inhibiting the chemokine-induced cell death. Altogether, data further support a role of CXCR2 axis in ALS etiopathogenesis and confirm its pharmacological modulation as a candidate therapeutic strategy.

## 1. Introduction

Amyotrophic lateral sclerosis (ALS) is a debilitating condition characterized by the degeneration of motor neurons (MNs) in the primary motor cortex and spinal cord, resulting in progressive muscle weakness and death within 2–5 years [1,2]. Most of the cases (90%) are sporadic (SALS) without a family history, while the remaining 10% are familial (familial ALS, FALS) mainly inherited in a dominant manner [1,3]. Disease-causing mutations in the Cu/Zn superoxide dismutase type-1 (*SOD1*) gene are common in ALS and account for both FALS and SALS, explaining approximately 12–20% of the familial and 1–2% of the sporadic cases [4,5]. The clinical presentation of SALS and FALS are similar, and treatment options remain mainly supportive so far. Indeed, the two current FDA-approved drugs, i.e., the anti-excitotoxic Riluzole (Rilutek) and the antioxidant Edaravone are able to prolong the lifespan of patients by only a few months and counteract disease progression without a real resolutive outcome [6,7].

The pathogenic process underlying ALS neurodegeneration is still not fully determined, although described alterations primarily consist of aberrant RNA metabolism, neuroinflammation, impaired protein homeostasis, mitochondrial dysfunction, excitotoxicity, and oxidative stress [8]. We have recently provided novel interesting clues about the role of the G-protein-coupled C-X-C motif chemokine receptor 2 (CXCR2) in ALS pathophysiology [9]. By using a bulk transcriptomic-based patients stratification approach and a following inter-species meta-analysis, we identified *CXCR2* mRNA as significant deregulated in human SALS motor cortex and SOD1-G93A mice at symptomatic stages as well, and therefore prioritized it as a candidate therapeutic target [8,9,10,11,12,13]. We further observed an increased immunoreactivity of the CXCR2 receptor in neuronal cell bodies and axons from ALS motor cortex [9], and functionally showed that receptor inhibition prevent inducible pluripotent stem cells (iPSC)-derived MNs degeneration in vitro and improve SOD1-G93A mice muscular functions in vivo, delaying the onset of neuromuscular decline by four weeks [9].

CXCR2 is a G-protein-coupled receptor, mainly involved in neuroinflammation, self-defense mechanisms against apoptotic cell death, chemotaxis of oligodendrocyte precursors during development, and modulation of synaptic transmission [14,15,16,17]. Its main ligand is Interleukin-8 (CXCL8/IL-8) that along with the functional murine homologues CXCL1 (also named growth-regulated GRO protein alpha, GROα/KC) and CXCL2 (or macrophage inflammatory protein 2-alpha, MIP2α) represent the primary chemokine system mediating neutrophil recruitment during both acute and chronic inflammation [18]. Significant high levels of CXCL8 have been previously reported in cerebrospinal fluid, blood, and serum collected from ALS patients [19,20,21,22,23,24,25,26], suggesting this axis may mediate neuroinflammation in ALS at both central and peripheral level. 

Deregulation of the CXCR2/ligands signaling has been previously described for further neuropathological diseases (traumatic brain injury, multiple sclerosis, ischemia, Alzheimer’s disease, neuropathic pain) [14,27], but the biological significance of this alteration in ALS remains not fully explained. 

In the present work, we investigated the expression and localization of both CXCR2 and CXCL8 in spinal cord *specimens* from control and ALS patients, and inspected the biological and functional role of the CXCR2/ligands axis in murine NSC-34 motor neuron-like cells expressing human wild-type (WT) or mutant G93A-SOD1. 

## 2. Materials and Methods

### 2.1. Transcriptomic Profiling 

For this study, we refer to a previously described transcriptome dataset [11,28] deposited in ArrayExpress (http://www.ebi.ac.uk/arrayexpress/, accessed on 30 January 2022) with the accession number E-MTAB-8635 (https://www.ebi.ac.uk/arrayexpress/experiments/E-MTAB-8635/, accessed on 30 January 2022). The dataset consists of the expression profiles of spinal cord samples from SALS (*n* = 30) and control (*n* = 10) subjects obtained with 4 × 44 K Whole Human Genome Oligo expression microarrays containing 41,093 probes (Agilent Technologies, Santa Clara, CA, USA). A detailed description of the subject characteristics (origin, source code, age, gender, race, disease state, survival time from diagnosis date and post-mortem interval) and experimental procedures was previously reported [11,28]. The E-MTAB-8635 dataset has been previously queried to investigate the role of splicing players deregulation in sporadic ALS [28], but not to explore the CXCR2/CXCL8 axis. Raw intensity signals from samples hybridization were thresholded to 1, log2-transformed, normalized, and baselined to the median of all samples using GeneSpring GX (Agilent Technologies, Santa Clara, CA, USA). Values from multiple probe signals targeting the same gene were collapsed to create a gene-level analysis and filtered to focus on CXCR2/CXCL8. The statistical analysis between CTRL and SALS was performed using *t*-test followed by Tukey post hoc test to identify significant variation between groups. 

### 2.2. Fluorescent Immunohistochemistry 

Post-mortem frozen sections (thick 10 μm) of lumbar (L1) spinal cord of control and ALS patients were obtained as described elsewhere [11]. Subject characteristics of patient samples used in the present study are reported in Supplementary Table S1. 

For immunofluorescence, paraformaldehyde (PFA)-fixed spinal cord *specimens* were permeabilized with Triton-X (0.2%) in PBS for 10 min, blocked with 10% NGS (normal goat serum), 2% BSA (bovine serum albumin), and 0.1% of Triton-X in PBS for 1 h, and incubated with anti-CXCR2 (rabbit ab14935, Abcam, Cambridge, UK), anti-Choline acetyltransferase (CHAT; mouse MA5-31383, Thermo Fisher Scientific, Waltham, MA, USA or rabbit ab137349 Abcam, Cambridge, UK), and anti-CXCL8 (mouse M801, Thermo Fisher Scientific, Waltham, MA, USA) primary antibodies at 4° overnight. TRITC and FITC-conjugated secondary antibodies (Goat anti-rabbit 111-025-003 and Goat anti-mouse 115-095-003, Jackson ImmunoResearch Laboratories Inc., Baltimore, PA, USA) were incubated for 1 h at room temperature shielded from light. Slides were washed 3 times in PBS after every step, and mounted with glycerol mounting medium supplemented with 4′,6-diamidino-2-phenylindole (DAPI). After drying, slides were analyzed with a Nikon A1 confocal inverted microscope equipped with a Plan Apochromat lambda 60×/1.4 oil immersion lens (Nikon, Tokyo, Japan). 

### 2.3. Cell Cultures 

Mouse MN-like hybrid NSC-34 cell line (provided by Dr. Cinzia Volontè from the National Research Council, Institute for Systems Analysis and Computer Science “Antonio Ruberti”, Cellular Neurobiology Unit, IRCCS Fondazione Santa Lucia, Rome, Italy) [29] was stably transfected with the pTet-ON plasmid (Clontech, Palo Alto, CA, USA), carrying the reverse tetracycline controlled transactivator used to induce the expression of human wild-type (WT) or mutant G93A SOD1 (hSOD1-G93A) cDNAs, as previously described [30,31]. Cells were grown in a medium composed by 1:1 DMEM and Ham’s F-12K Nutrient Medium (Sigma-Aldrich, Munich, Germany), 15 mM HEPES (Sigma-Aldrich, Munich, Germany), 10% of heat-inactivated FBS (Invitrogen, New York, NY, USA), 100 U/mL penicillin, and 100 μg/mL streptomycin (Sigma-Aldrich, Munich, Germany). Cell cultures were incubated at 37 °C in 5% CO_2_. The addition of 2 µg/mL doxycycline (Sigma-Aldrich, Munich, Germany) for the last 24 h of culture was used to induce the expression of human WT or mutant (G93A) SOD1. 

### 2.4. Cell Viability 

Cell viability was assessed using the colorimetric reagent-based MTT cell proliferation kit I, based on 3-[4,5-dimethylthiazol-2-yl]-2,5-diphenyltetrazolium bromide (Roche Diagnostics, Mannheim, Germany) salt, as previously described [32]. Cells were cultured into 96-well plates at a density of 1 × 10^4^ cells/well in 100 μL of medium for 24 h. The day after doxycycline induction, cells were treated with increasing concentration of CXCL1/GROα (SRP4210, Sigma-Aldrich, Munich, Germany) and CXCL2/MIP2α (SRP4251, Sigma-Aldrich, Munich, Germany) for 24 h. Subsequently, 0.5 mg/mL of MTT was added to each well and incubated for 4 h at 37 °C. The reaction was stopped with 100 μL of solubilization solution, then formazan was measured spectrophotometrically (550–600 nm) using a microplate reader (Bio-Rad Laboratories, Hercules, CA, US). Six replicate wells were used for each group. Controls included untreated cells, and medium alone was used as a blank. The minimum effective dose of agonists in vitro to produce significant cell death, i.e., GROα (1 ng/mL) and MIP2α (100 nM), was chosen and used for following experiments alone or in combination with 10 μM reparixin (MedChem Express, Monmouth Junction, NJ, USA) as previously described [9]. 

To perform cell counting, NSC-34 WT and NSC-34 G93A cells were seeded into 24 well plates (25,000 cells for each well) and cultured for 24 h at 37 °C in 5% CO_2_ in the medium previously described. The day after, hSOD1 expression was induced by adding 2 µg/mL doxycycline for 24 h. Then, the culture medium was replaced with fresh medium containing GROα (1 ng/mL) or MIP2α (100 nM) in the presence or absence of reparixin (Rep) (10 μM). After 24 h, cells were trypsinized and centrifugated at 10,000× *g* for 7 min at 4 °C. The pellet was resuspended in 1 mL of fresh medium, then 100 μL of cell suspension were added to a trypan blue solution for cell counting in a Bürker chamber.

### 2.5. Western Blot Analysis

Proteins were extracted from total cells lysate with RIPA buffer (Thermo Fisher Scientific, Paisley, UK) supplemented with phosphatase and protease inhibitors (Roche Diagnostics, Monza, Italy), homogenized by a Teflon-glass homogenizer and then sonicated by an ultrasonic probe, followed by centrifugation at 10,000× *g* for 10 min at 4 °C. Quant-iT Protein Assay Kit (Invitrogen, NY, USA) was used to determine protein concentration for each sample as previously described [33]. About 25 μg of protein homogenate was diluted in 2X Laemmli buffer (Invitrogen, NY, USA), heated at 70 °C for 10 min, separated on a Biorad Criterion XT 4–15% Bis-tris gel (Invitrogen, NY, USA) by electrophoresis and then transferred to a nitrocellulose membrane (Invitrogen). The transfer was monitored by a pre-stained protein molecular weight marker (BioRad Laboratories, Hercules, CA, USA). The nitrocellulose membranes were firstly incubated with Odyssey Blocking Buffer (Li-Cor Biosciences, Lincoln, Nebraska) and subsequently with specific primary antibodies: anti-CXCR2 (rabbit ab217314, Abcam, Cambridge, UK), anti-BAX (mouse sc-20067, Santa Cruz Biotechnology, Inc., Dallas, TX, USA), anti-BCL2 (mouse sc-509, Santa Cruz Biotechnology, Inc., Dallas, Texas), and anti-β-actin (mouse sc-47778, 1:500, Santa Cruz Biotechnology, Inc., Dallas, Texas).

The goat anti-rabbit IRDye 800CW (#926-32211; Li-Cor Biosciences) and the goat anti-mouse IRDye 680CW (#926-68020D; Li-Cor Biosciences, Lincoln, NE, USA) secondary antibodies were diluted at 1:20,000 and 1:30,000, respectively. Blots were scanned with an Odyssey Infrared Imaging System (Odyssey), and densitometric analysis was performed at non-saturating exposures using the ImageJ software (Version 1.53t, NIH, Bethesda, MD, USA) as previously described [34,35]. β-actin was used as loading control for normalization.

### 2.6. Immunocytofluorescence

NSC-34 cells expressing human WT or SOD1-G93A were cultured on glass cover slips, fixed in 4% PFA in PBS for 15 min at room temperature, permeabilized with Triton X-100 (0.2%), blocked with BSA (0.1%) in PBS for 1 h at room temperature and probed with the anti-CXCR2 (rabbit ab14935, Abcam, Cambridge, UK) or anti-cleaved-caspase-3 Asp 175 (rabbit #9661, Cell Signaling) primary antibodies overnight. Subsequently, samples were incubated with the Alexa Fluor 488 goat anti-rabbit antibody for 1 h at room temperature shielded from light. DAPI was used to stain the nuclei (#940110 Vector Laboratories). Images were captured with a Nikon A1 confocal inverted microscope equipped with a Plan Apochromat lambda 60×/1.4 oil immersion lens (Nikon, Tokyo, Japan). Fluorescence was quantified by extrapolating the mean intensity of FITC channel from multiple regions of interest (ROI), normalized to the background by using the NIS-Elements AR (Advanced Research) software (version 4.60).

### 2.7. Statistical Analysis

Data are reported as the mean ± standard error of the mean (SEM). *T*-test and one-way analysis of variance (ANOVA) were used to compare differences among groups. Tukey-Kramer post hoc test was applied to assess the statistical significance (*p* ≤ 0.05). All statistics were run using the Prism 5.0a (GraphPad Software Inc., La Jolla, CA, USA) software packages.

## 3. Results

### 3.1. CXCR2/CXCL8 Expression in Control and Sporadic ALS Spinal Cord Samples

Previous transcriptome profiling, qRT-PCR and immunohistochemistry experiments revealed a significant upregulation of CXCR2 in human sporadic ALS motor cortex compared to control, and its main localization in both somas and axons of cortical neurons [9,11]. Here, we focused on lumbar (L1) spinal cord *specimens* from the same cohort of patients, and analyzed *CXCR2* and its main ligand *CXCL8* at both mRNA and protein levels.

**Figure 1:**
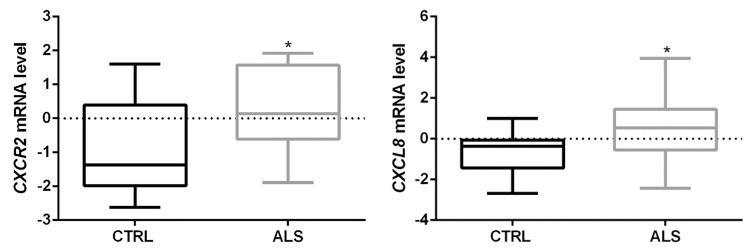
CXCR2 and CXCL8 mRNAs are upregulated in spinal cord samples of ALS patients. Transcriptomic data showed a statistically significant increase of both *CXCR2* and *CXCL8* mRNA expression levels in ALS spinal cord compared to CTRL. Data are extrapolated from the dataset E-MTAB-8635, and analyzed as described in the Materials and Methods section. Tukey-Kramer post hoc test: * *p* < 0.05 ALS vs. CTRL.

Gene-level transcriptional analysis (E-MTAB-8635 dataset) showed a significant increase of both *CXCR2* and *CXCL8* mRNA in ALS spinal cord tissue compared to controls (* *p* < 0.05 ALS vs. CTRL) (Figure 1). In addition, we found an association trend between *CXCR2* higher mRNA levels and short survival (Kaplan–Meier curves in Supplementary Figure S1).

**Figure 2:**
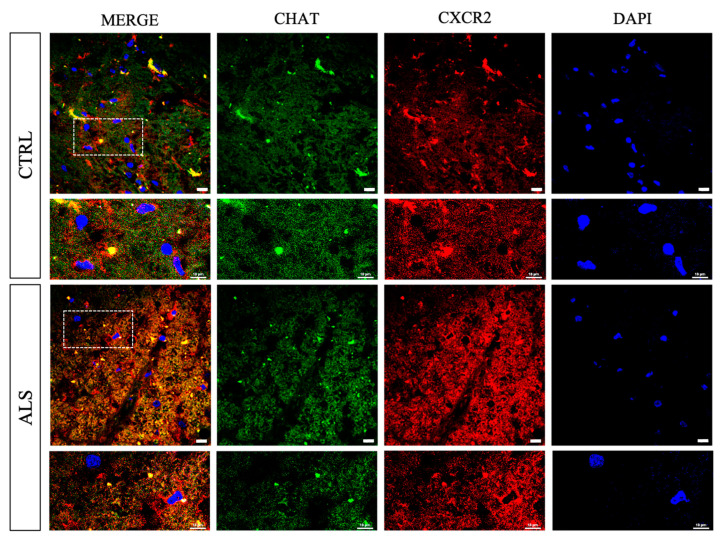
CXCR2 distribution in spinal cord anterior horns of control and ALS patients. Immunofluorescence analyses were used to investigate localization of CXCR2 immunoreactivity in the spinal cord ventral horns (in correspondence to CHAT^+^ neurons). Representative photomicrographs show CXCR2 immunoreactivity in spinal cord ventral horn regions of control and ALS patients examined under a Nikon A1 confocal inverted microscope equipped with a Plan Apochromat lambda 60×/1.4 oil immersion lens (Nikon, Tokyo, Japan). Scale bar 10 μm.

**Figure 3:**
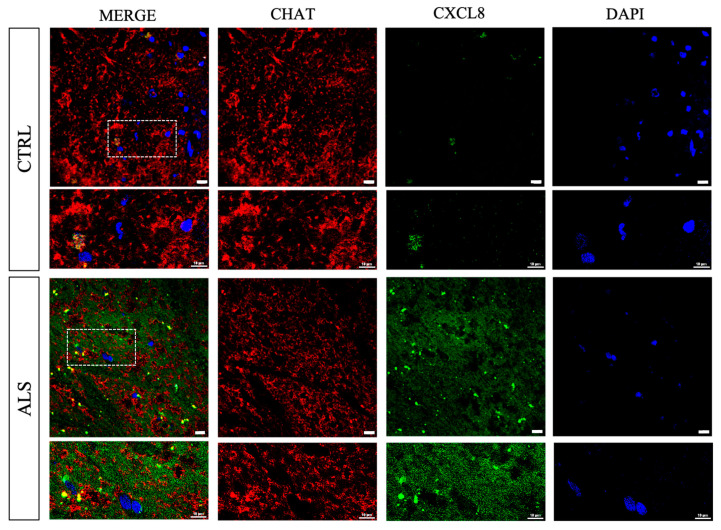
CXCL8 distribution in spinal cord anterior horns of control and ALS patients. Immunofluorescence analyses were used to investigate localization of CXCL8 immunoreactivity in the spinal cord ventral horns (in correspondence to CHAT^+^ neurons). Representative images show CXCL8 immunoreactivity in spinal cord ventral horn regions of control and ALS patients examined under a Nikon A1 confocal inverted microscope equipped with a Plan Apochromat lambda 60×/1.4 oil immersion lens (Nikon, Tokyo, Japan). Scale bar 10 μm.

Double staining with anti-CXCR2/CHAT antibodies revealed a substantial CXCR2 immunoreactivity in spinal anterior horns in correspondence to CHAT^+^ neurons, which was visibly increased in ALS samples (Figure 2). In the same region, CXCL8 immunoreactivity was mainly found in ALS (Figure 3).

### 3.2. CXCR2 Activation by GROα and MIP2α Pro-Inflammatory Chemokines Induces Dose-Dependent Cell Death in NSC-34 Cells Overexpressing hSOD1-G93A

**Figure 4:**
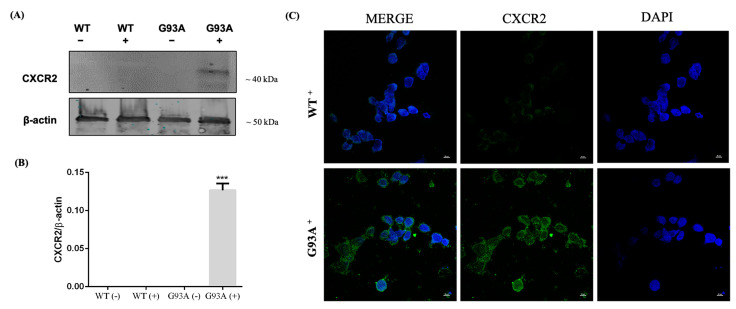
CXCR2 expression in WT and SOD1-G93A NSC-34 cell line. (**A**) Representative immunoblot of CXCR2 signals obtained using 25 μg of cell homogenate from WT and SOD1-G93A NSC-34 cell line before (−) or after (+) induction by doxycycline. (**B**) The bar graph shows representative results from three independent experiments. Relative signal density was quantified using the ImageJ software (Version 1.53t). Protein levels were expressed as arbitrary units obtained after normalization to β-actin, which was used as loading control. Data are expressed as mean ± SEM (*** *p* < 0.001 vs. WT^+^). (**C**) Immunofluorescence experiments showing CXCR2 immunoreactivity in WT and SOD1-G93A NSC-34. Nuclei were counterstained with DAPI. Photomicrographs are representative of randomly selected fields and scanned by Nikon Ti Eclipse inverted microscope. Scale bar 10 μm.

To explore the biological role of CXCR2 axis in ALS, we used an in vitro motor neuron-like model by using the mouse hybrid cell line NSC-34 overexpressing wild type (WT) and mutant human SOD1 (hSOD1-G93A) [30,31]. Western blot and immunofluorescence experiments were carried out to examine the expression of the receptor in both WT and mutant hSOD1-G93A cell line. Interestingly, we observed a consistent genotype-specific expression of CXCR2 in the activated NSC-34 cells carrying the mutated hSOD1 construct (*** *p* < 0.001 vs. WT^+^), while the same was faintly detected in WT cells (Figure 4). 

**Figure 5:**
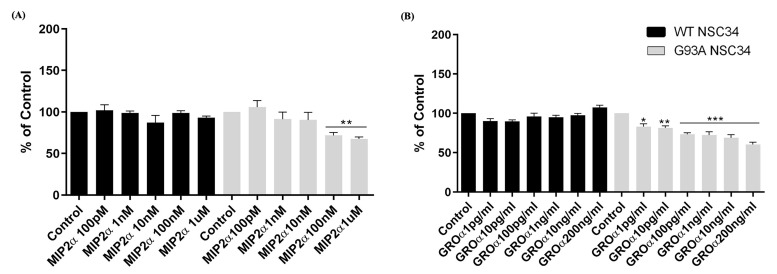
Dose dependent cell death of SOD1-G93A NSC-34 cells following MIP2α and GROα treatment. Analysis of WT and SOD1-G93A NSC-34 cell viability after exposure with different concentrations of MIP2α (**A**) and GROα (**B**) for 24 h assessed by MTT. Normal growth medium cultured cells were used as controls. Results are representative of three independent experiments and values are expressed as percentage of control (* *p* < 0.05, ** *p* < 0.01 or *** *p* < 0.001 vs. Control as determined by one-way ANOVA followed by Tukey-Kramer post hoc test).

To further investigate the contribution of CXCR2 activation by endogenous ligands in ALS neuronal depletion, we exposed both WT and mutant hSOD1-G93A NSC-34 cells to increasing concentrations of the two murine functional homologs of CXCL8, i.e., MIP2α (100 pM, 1 nM, 10 nM, 100 nM, 1 µM) and GROα (1 pg/mL, 10 pg/mL, 100 pg/mL, 1 ng/mL, 10 ng/mL, 100 ng/mL), and assessed cellular viability after 24 h. Both GROα and MIP2α treatments determined a significant reduction of cellular viability in a dose-dependent manner in hSOD1-G93A NSC-34 cells but not in WT, compared to untreated cells (** *p* < 0.01 or *** *p* < 0.001 vs. Control) (Figure 5). The minimum effective dose of both ligands to induce significant cell death in hSOD1-G93A NSC-34 cells (1 ng/mL GROα and 100 nM MIP2α) was chosen for the following experiments. 

We investigated the CXCR2 expression following ligand treatment in the G93A^+^ background, and observed an increase of CXCR2 immunoreactivity 24 h after MIP2α or GROα incubation (Supplementary Figure S2).

**Figure 6:**
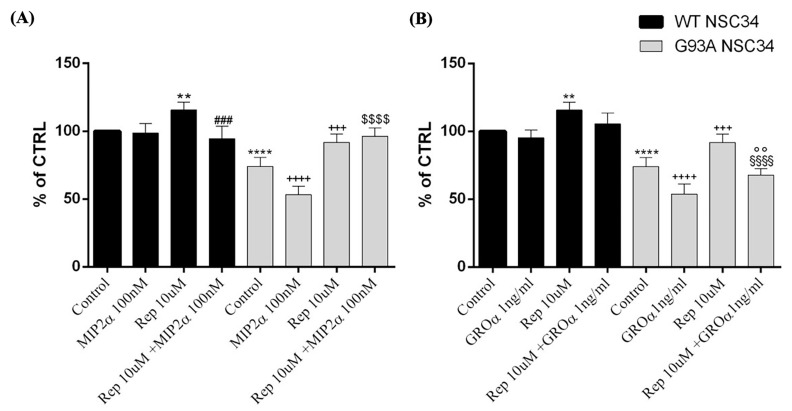
Effects on cellular viability after CXCR2 pharmacological blockade by reparixin treatment in SOD1 NSC-34 cells. WT and SOD1-G93A NSC34 cells cultured in normal growth medium (Control), exposed to MIP2α (**A**) or GROα (**B**) for 24 h, in the presence or absence of reparixin (Rep). Results are representative of at least three independent experiments and values are expressed as percentage of control (** *p* < 0.01 or **** *p* < 0.0001 vs. control WT, ^###^ *p* < 0.001 vs. Rep WT, ^+++^ *p* < 0.001 or ^++++^ *p* < 0.0001 vs. control G93A, ^$$$$^ *p* < 0.0001 vs. MIP2α, ^§§§§^ *p* < 0.0001 vs. Rep G93A, ^°°^ *p* < 0.01 vs. GROα as determined by one-way ANOVA followed by Tukey–Kramer post hoc test).

To investigate whether the in vitro neurotoxicity of GROα and MIP2α was specifically mediated by CXCR2, we tested reparixin, a non-competitive allosteric inhibitor of this receptor. Both MTT analysis (Figure 6) and cell counting (Supplementary Figure S3) showed that treatment with reparixin (10 μM) counteracted the toxicity of both 1 ng/mL GROα and 100 nM MIP2α, significantly inhibiting chemokine-induced cell death and playing a significant neuroprotective role in hSOD1-G93A NSC-34 cells.

### 3.3. Activation of CXCR2 Axis by MIP2α Triggers Apoptosis in hSOD1-G93A NSC-34 Cells

**Figure 7:**
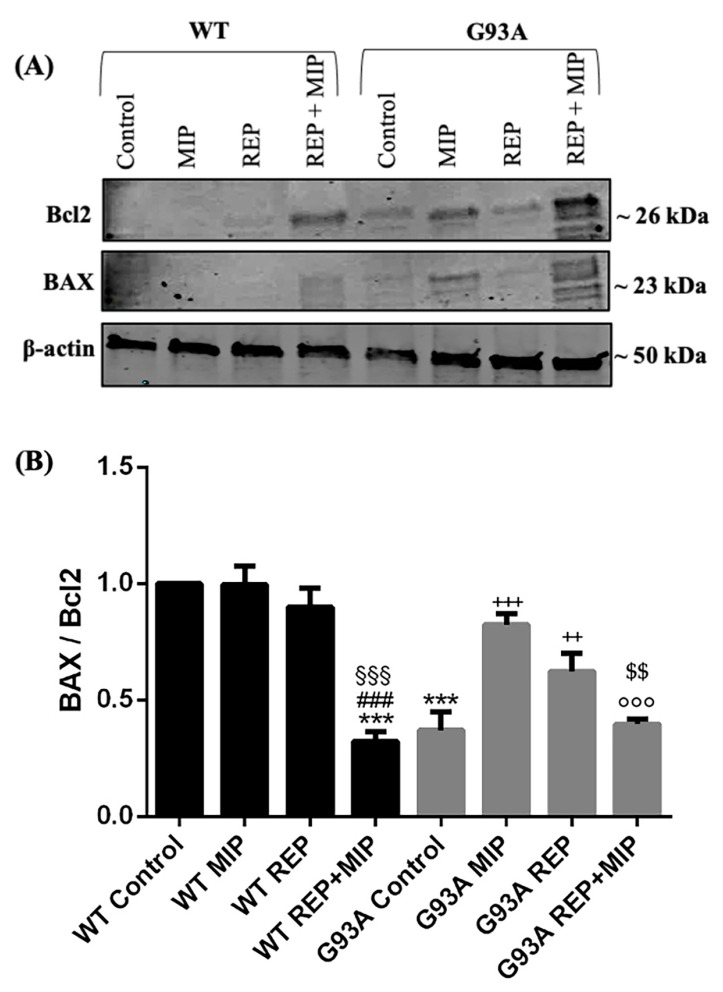
BAX and BCL2 expression in WT and SOD1-G93A NSC-34 cells after CXCR2 activation by MIP2α and antagonism by reparixin. (**A**) Representative immunoblot signals obtained by BAX and BCL2 antibodies from WT and SOD1-G93A NSC-34 cells cultured in normal growth medium (Control), with the addition of MIP2α alone or in combination with reparixin (Rep). (**B**) The bar graph shows results from three independent experiments. Relative signal density was quantified using the ImageJ software (Version 1.53t). The protein levels were expressed as arbitrary units obtained after normalization to β-actin, which was used as loading control. Data are expressed as mean ± SEM (*** *p* < 0.001 vs. Control WT; ^§§§^ *p* < 0.001 vs. MIP2α WT; ^###^ *p* < 0.001 vs. REP WT; ^++^ *p* < 0.01 or ^+++^ *p* < 0.001 vs. Control G93A; ^$$^ *p* < 0.01 vs. REP G93A; ^°°°^ *p* < 0.001 vs. MIP2α G93A, as determined by one-Way ANOVA followed by Tukey post hoc test).

To further explore the molecular mechanisms underlying cell death induced by CXCR2 activation, we examined by Western blot analysis the expression of two players involved in apoptosis, the pro-apoptotic BAX and the anti-apoptotic BCL2, following MIP2α and reparixin treatment. Densitometric analysis revealed that CXCR2 activation by MIP2α triggers apoptosis in hSOD1-G93A NSC-34 cells, prompting a significant upregulation of the ratio BAX/BCL2, while the simultaneous antagonism by reparixin significantly reduced the ratio compared to the agonist alone (Figure 7). These data are consistent with the effects produced on cellular viability. No significant effect was observed for WT cells, with the exception of co-treatment with MIP2α and reparixin, which elicited a downregulation of the BAX/BCL2 ratio.

**Figure 8:**
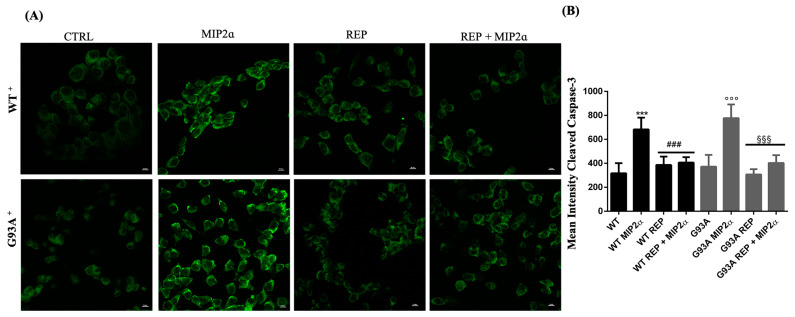
Cleaved-caspase-3 staining in WT and SOD1-G93A NSC-34 cells after MIP2α and reparixin incubation. (**A**) Representative images showing the immunoreactivity of cleaved caspase-3 in WT and SOD1-G93A NSC-34 cells following treatment with MIP2α alone or in combination with reparixin (Rep). (**B**) The bar graph shows the mean fluorescence intensity of cleaved caspase-3 quantified extrapolating the intensity of FITC channel from multiple regions of interest (ROI) normalized to the background by using the NIS-Elements AR (Advanced Research) software (version 4.60). *** *p* < 0.001 versus control WT, ^###^ *p* < 0.001 versus MIP2α WT, ^°°°^ *p* < 0.001 versus control G93A, ^§§§^ *p* < 0.001 versus MIP2α G93A.

To further consolidate these results, we analyzed by fluorescent immunocytochemistry the levels of cleaved caspase-3. Results obtained showed an increased cleaved caspase-3 immunoreactivity in MIP2ɑ-incubated cells, while levels were decreased in combination with reparixin (Figure 8).

## 4. Discussion

A plethora of molecular mechanisms have been proposed to account for neuronal damage in ALS, including aberrant RNA metabolism, impaired protein homeostasis, mitochondrial dysfunction, excitotoxicity, and oxidative stress, likely arising from a combination of environmental and genetic risk factors [28,36,37,38]. Increasing evidence are suggesting that neuroinflammation plays a key role in neuronal degeneration in both SALS and FALS [25,39]. Chronically activated astrocytes, microglia, and infiltrating T cells represent prominent pathological features founded at sites of motor neuron injury on end-stage pathology in both patients and animal models [39,40]. In addition, dysregulation in circulating lymphocyte and monocyte populations, as well as altered levels in inflammatory cytokines, chemokines, growth factors (such as VEGF, IFN-γ, TNF-α, IL-1β, IL-6, and IL-10) and their receptors have been reported at different disease stages [22,41].

In the present work, we provide further evidence supporting a role of the G-protein-coupled receptor CXCR2 axis in ALS pathophysiology. The expression of this receptor, mainly involved in mediating inflammatory response, has been previously reported in projections of cerebral cortex neurons, hippocampus, cerebellum [15], human cortical motor neurons (pyramidal cells in layer V) [9], neutrophils [42], monocytes [43], T-lymphocytes, mast cells [44,45], fibroblasts [46], and endothelial cells [47]. CXCR2 expression was also detected in mice spinal neurons (NeuN^+^ cells) [27], and here we show for the first time that the receptor is expressed in human spinal cord anterior horns, and that its level significantly increases in ALS terminal stages (Figure 1 and Figure 2). Interestingly, by correlating the CXCR2 mRNA level to disease progression, we found a trend of association between *CXCR2* higher level and short survival (Kaplan–Meier curves in Supplementary Figure S1). 

Activation of CXCR2 is carried out by the binding of its endogenous ligands, among which Interleukin-8 (CXCL8) represents the main one [14,18]. Different studies have reported a significant increase of CXCL8 during ALS development and progression, both at systemic (e.g., blood, serum) and central (CSF or spinal cord tissue) levels [19,20,21,22,23,24,25,26]. Accordingly, we confirmed a significant increase of CXCL8 level in the spinal cord of sporadic ALS patients and reported its expression in spinal ventral horns (Figure 1 and Figure 3). 

To further explore the involvement of CXCR2 axis in ALS, we investigated its functional biological role in murine hybrid neuroblastoma–spinal cord NSC-34 cells overexpressing human WT and mutant G93A-SOD1. These cells represent a widely used in vitro model to study MNs in an immortalized system, and constitutively express many phenotypic features of primary motor neurons, such as neurofilament triplet proteins expression, the generation of action potentials, synthesis/storage of acetylcholine, and sensitivity to glutamate insult [31]. Moreover, preliminary observations suggested that in several biological scenarios characterized by mutated SOD1, there is an overall deregulation of the CXCR2/CXCL8 pathway. Indeed, a significant upregulation of CXCR2 ligands emerged in cervical MNs isolated from SOD1 familiar ALS cases compared to control, as well as in fully differentiated iPSC-derived MNs carrying mutated SOD1 (Supplementary Table S2). Moreover, a previous time-course metanalysis of different transcriptomic profiles revealed a significant increase of *Cxcr2* in the spinal cords of SOD1-G93A mice at symptomatic/terminal stages (100–120 days of age) (Supplementary Figure S4) [10,48].

Consistent with these findings, we observed a genotype-specific expression of CXCR2 in hG93A-SOD1 NSC-34 cells, but not in WT hSOD1 (Figure 4). In this regard, a pathological increase of this receptor may provide a paracrine or autocrine milieu, which may enable inflammatory/immune responses inconsistent with healthy neural function [49,50]. Indeed, receptor activation by GROα and MIP2α, two murine endogenous ligands of Cxcr2 and functional homologs of CXCL8, triggered apoptosis and reduced cellular viability in a dose dependent manner (Figure 5, Figure 6, Figure 7 and Figure 8). Moreover, treatment with reparixin, a non-competitive allosteric CXCR2 inhibitor, counteracted the effects of GROα and MIP2α, inhibiting their toxic effects (Figure 6, Figure 7 and Figure 8). It is noteworthy that all these findings are consistent with previous observations establishing a neuroprotective effect of reparixin against MIP2α-induced toxicity in rodent-based motor neuronal primary cultures [51], against growth-factor deprivation-induced apoptosis in human degenerating iPSC-derived motor neurons, and in SOD1-G93A transgenic mice [9]. 

## 5. Conclusions

Although further studies are still necessary to completely elucidate the contribution of CXCR2/ligands in ALS pathogenesis and to define the impact of this signaling pathway in motor neuronal selective degeneration, our data further support a role of this axis in ALS pathogenesis and confirm its pharmacological modulation as a candidate therapeutic strategy against ALS.

## Data Availability

Transcriptional data are available at EBI ArrayExpress database with the accession number E-MTAB-8635 (https://www.ebi.ac.uk/arrayexpress/experiments/E-MTAB-8635/).

## Supplementary Materials

The supplementary file can be downloaded at: https://www.mdpi.com/article/10.3390/cells12141813/s1 and include Supplementary Figure S1: Kaplan-Meier curve correlating the ALS patients’ survival with the CXCR2 mRNA level in spinal cord; Supplementary Figure S2: CXCR2 immunoreactivity in activated NSC-34 cells with the G93A background after MIP2α and GROα incubation; Supplementary Figure S3: Number of viable cells after CXCR2 activation or pharmacological blockade by reparixin treatment in NSC-34 WT and SOD1-G93A cell lines; Supplementary Figure S4: Time-course analysis of Cxcr2 mRNA levels in spinal cord of SOD1-G93A mice at different stages of disease; Supplementary Table S1: Phenotypic characteristics of subjects (race, gender, age, disease state, survival characteristics and post-mortem interval) used in the present study; Supplementary Table S2: CXCR2 ligands differentially expressed in SOD1-mutant MNs from transcriptomic datasets.
