# Supplementary material for: CXCR2 Is Deregulated in ALS Spinal Cord and Its Activation Triggers Apoptosis in Motor Neuron-Like Cells Overexpressing hSOD1-G93A
Source: Cells. 2023 Jul 9;12(14):1813. doi: 10.3390/cells12141813 (PMC10377984; doi:10.3390/cells12141813)
Supplement: Supplementary file 1 [file cells-12-01813-s001.zip › cells-2254664-supplementary.pdf]

Supplementary file

CXCR2 is deregulated in ALS spinal cord and its activation triggers apoptosis in motor neuron-like cells overexpressing hSOD1-G93A

Valentina La Cognata[1§], Agata Grazia D'Amico[2§], Grazia Maugeri[2], Giovanna Morello[1], Maria Guarnaccia[1], Benedetta Magrì[2], Eleonora Aronica[3], Velia D'Agata[2], Sebastiano Cavallaro[1]**

1 Institute for Biomedical Research and Innovation, National Research Council, Catania, Italy; valentina.lacognata@irib.cnr.it; giovanna.morello@irib.cnr.it; maria.guarnaccia@cnr.it; sebastiano.cavallaro@cnr.it

2 Section of Human Anatomy and Histology, Department of Biomedical and Biotechnological Sciences, University of Catania, Catania, Italy; agata.damico@unict.it; graziamaugeri@unict.it; benedetta.magri@phd.unict.it, vdagata@unict.it

3 Department of (Neuro) Pathology, Amsterdam UMC, University of Amsterdam, Amsterdam Neuroscience, Meibergdreef 9, 1105 Amsterdam, the Netherlands; e.aronica@amsterdamumc.nl

§ VLC e AGD contributed equally.

***** Correspondence: sebastiano.cavallaro@cnr.it; vdagata@unict.it

Supplementary Figure S1

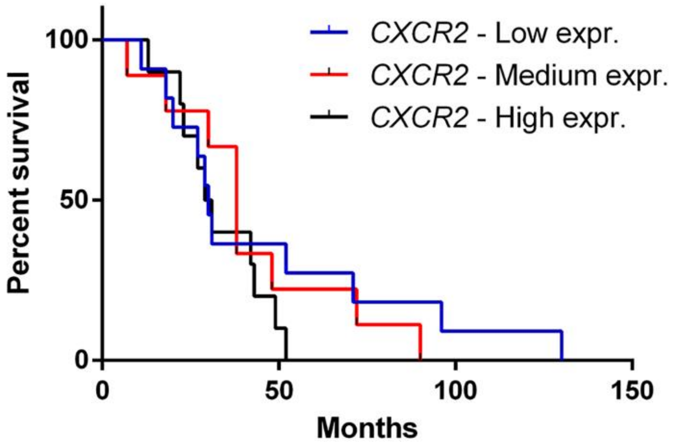

Kaplan-Meier curve correlating the ALS patients' survival with the *CXCR2* mRNA level in spinal cord. The figure correlates the survival (in months) of ALS patients [1,2] with the low, medium or high normalized expression levels of *CXCR2* mRNA in spinal cord. Expression values were divided in three percentiles. ALS patients with i) *CXCR2* low expression: n.= 11; ii) *CXCR2* medium expression: n.= 9; iii) *CXCR2* high expression n.= 10.

Supplementary Figure S2

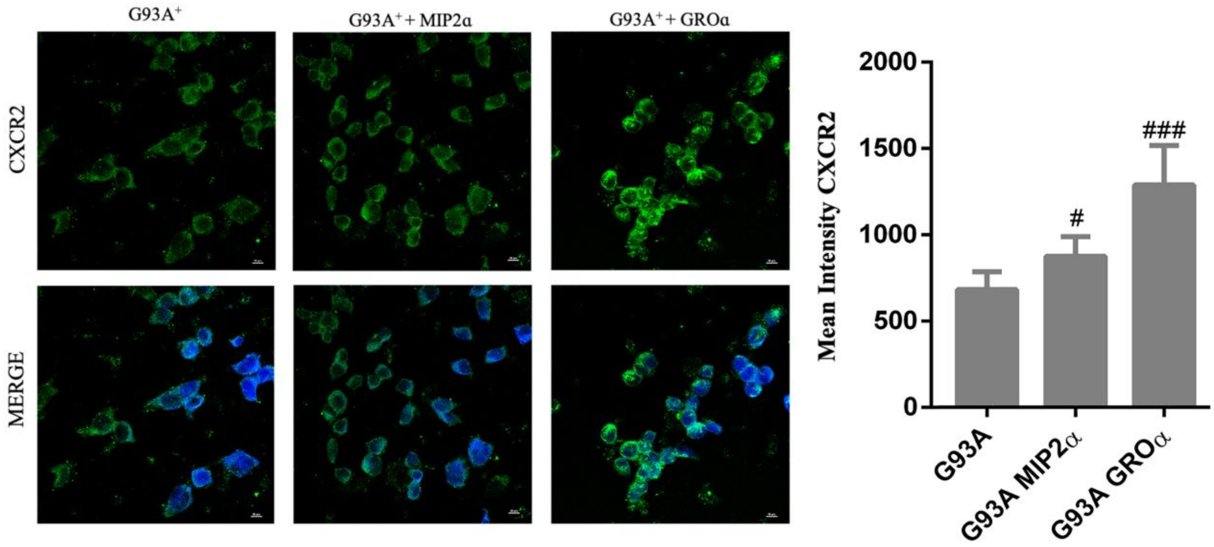

CXCR2 immunoreactivity in activated NSC-34 cells with the G93A background after MIP2α and GROα incubation. Immunofluorescence experiments showing the immunoreactivity of CXCR2 in SOD1-G93A NSC-34 cells after 24 hours of treatment with MIP2α (100 nM) or GROα (1 ng/ml). Images are representative of randomly selected fields scanned by Nikon Ti Eclipse inverted micro-scope. Scale bar 10 µm. Fluorescence was quantified extrapolating the mean intensity of FITC channel from multiple regions of interest (ROI) normalized to the background by using the NIS-Elements AR (Advanced Research) software. Data are expressed as Mean ± SEM (#p<0.05 vs control G93A, ###p<0.001 vs control G93A as determined by One-way ANOVA followed by Tukey–Kramer *post hoc* test).

Supplementary Figure S3

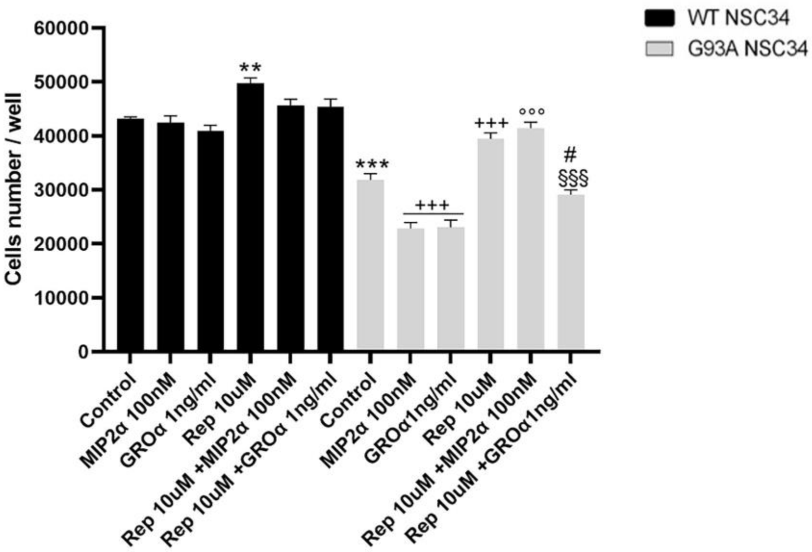

Number of viable cells after CXCR2 activation or pharmacological blockade by reparixin treatment in NSC-34 WT and SOD1-G93A cell lines. The bar graph shows the number of viable cells evaluated through trypan blue exclusion assay. Data are expressed as Mean ± SEM (**$p<0.01$ or ***$p<0.001$ vs Control WT, +++$p<0.001$ vs Control G93A, °°°$p<0.001$ vs MIP2α G93A, §§§$p<0.001$ vs GROα G93A, #$p<0.05$ vs Rep G93A, as determined by One-way ANOVA followed by Tukey–Kramer *post hoc* test).

Supplementary Figure S4

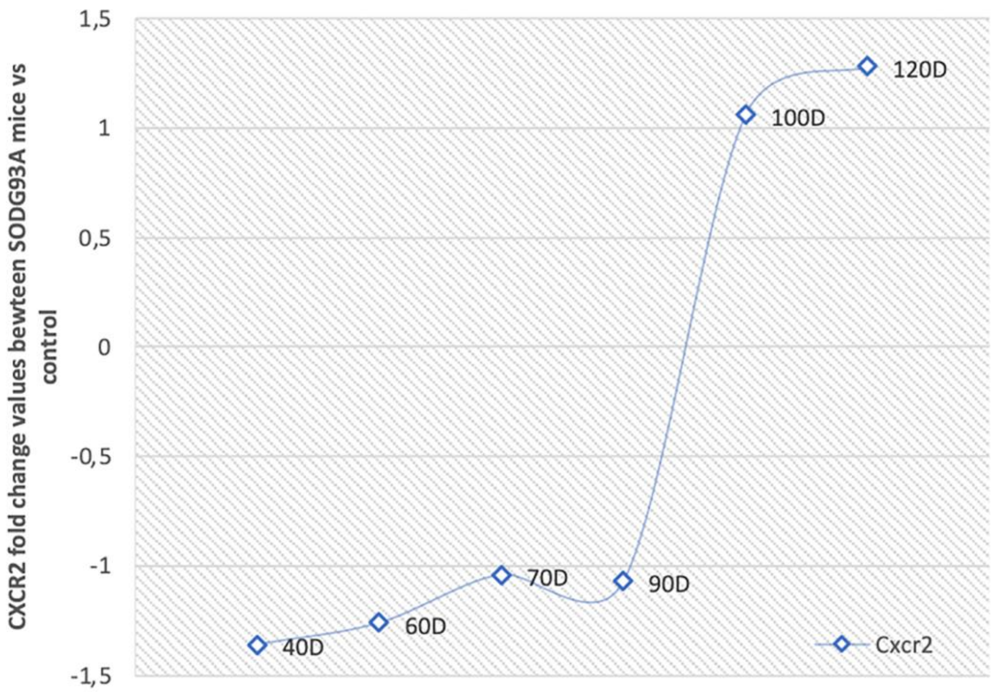

GeneSymbol	SOD1G93A vs control 40 days (GSE50642)	SOD1G93A vs control 60 days (GSE56926)	SOD1G93A vs control 70 days (GSE27933)	SOD1G93A vs control 90 days (GSE10953)	SOD1G93A vs control 100 days (GSE27933)	SOD1G93A vs control 120 days (GSE10953)
Cxcr2	-1,36	-1,07	-1,04	-1,07	1,06	1,28

Time-course analysis of *Cxcr2* mRNA levels in spinal cord of SOD1-G93A mice at different stages of disease. The figure illustrates the *Cxcr2* expression fold changes between SOD1-G93A mice versus littermate controls (y-axis) at different time points of disease state, i.e., pre-symptomatic (40-60 days), symptomatic (70–90 days), and terminal (100–120 days) (x-axis). For generating time-course analysis, we referred to following datasets: GSE50642 for 40 days old, GSE56926 for 60 days old, GSE27933 for 70- and 100-days old, and GSE10953 for both 90- and 120-days old mice, respectively. Each point represents the averaged fold change value of all probe sets representing the gene.

Supplementary Table 1

Phenotypic characteristics of subjects (race, gender, age, disease state, survival characteristics and post-mortem interval) used in the present study for IF experiments.

Source name	Individual	Race	Gender	Age	PMI (hours)	Disease state	Survival characteristics
**Sample 3**	#13 09-13-07	Caucasian	M	62	7,5	Control	n.a.
**Sample 4**	#16 09-13-07	Caucasian	F	71	9,5	Control	n.a.
**Sample 5**	#18 09-13-07	Caucasian	M	73	11	Control	n.a.
**Sample 24**	#54 09-13-07	Caucasian	M	51	8	SALS	Short-term survival
**Sample 30**	#66 09-13-07	Caucasian	M	54	3	SALS	Medium-term survival
**Sample 32**	#70 09-13-07	Caucasian	F	61	10	SALS	Medium-term survival

Patients code is the same reported in dataset E-MTAB-8635.

Supplementary Table 2

CXCR2 ligands differentially expressed in *SOD1*-mutant MNs from transcriptomic datasets.

	**GSE106382**	**GSE20589**
**Gene Symbol**	Fold Change (FALS SOD1/CTRL)	Fold Change (FALS SOD1/CTRL)
CXCL8	13,52	2,71
CXCL1	6,43	-
CXCL2	1,82	-

The table shows the mRNA expression of *CXCR2* ligands in SOD1-mutant iPSC-derived MNs (DataSet Accession ID: GSE106382) and laser-capture micro-dissected MNs isolated from FALS patients carrying SOD1 mutation (DataSet Accession ID: GSE20589) compared to healthy donors. For each gene, average fold change values of differential expression (p-value <0.05) between SOD1-linked FALS versus individual controls are shown. For each study, Gene Expression Omnibus DataSet Accession ID is reported.

References

1. Aronica, E.; Baas, F.; Iyer, A.; ten Asbroek, A.L.; Morello, G.; Cavallaro, S. Molecular classification of amyotrophic lateral sclerosis by unsupervised clustering of gene expression in motor cortex. *Neurobiol Dis* **2015**, *74*, 359-376, doi:10.1016/j.nbd.2014.12.002.
2. La Cognata, V.; Gentile, G.; Aronica, E.; Cavallaro, S. Splicing Players Are Differently Expressed in Sporadic Amyotrophic Lateral Sclerosis Molecular Clusters and Brain Regions. *Cells* **2020**, *9*, doi:10.3390/cells9010159.
